# Multi-lingual “Asthma APP” improves health knowledge of asthma among Australian First Nations carers of children with asthma

**DOI:** 10.3389/fped.2022.925189

**Published:** 2022-08-30

**Authors:** Lesley A. Versteegh, Anne B. Chang, Sharon Chirgwin, Fransisca P. Tenorio, Catherine A. Wilson, Gabrielle B. McCallum

**Affiliations:** ^1^Child Health Division, Menzies School of Health Research, Charles Darwin University, Darwin, NT, Australia; ^2^Centre for Children's Health Research, Queensland University of Technology, Brisbane, QLD, Australia; ^3^Department of Respiratory and Sleep Medicine, Queensland Children's Hospital, Brisbane, QLD, Australia; ^4^Clinical Innovation and Research, Northern Territory Health, NT, Australia

**Keywords:** First Nations, asthma, Indigenous, education, mHealth, mobile phones, digital health

## Abstract

**Background:**

Among Australian First Nations people, asthma is associated with worse morbidity and mortality than non-First Nations people. Improving the delivery of health education that is innovative and culturally relevant to linguistically diverse populations is needed. Digital platforms, such as mobile applications (APP), have the potential to improve evidence-based health education, particularly in settings where access to specialist services is limited and turnover of staff is high, such as in remote Australia. In response to consumer needs, we developed a multi-lingual Asthma APP from our existing asthma flipchart, with a “voice-over” in seven local First Nations languages and English, using a mixture of static and interactive formats. In this study, we evaluated (a) the functionality and usability of the APP with First Nations health professionals with and without asthma and (b) whether the APP improves health knowledge and understanding of asthma among First Nations carers of children with asthma.

**Methods:**

In total, 7 First Nations health professionals participated in semi-structured interviews prior to the evaluation with 80 First Nations carers of children with asthma from the Northern Territory and Queensland, Australia. Carers underwent pre- and post-education questionnaires (maximum score = 25), where the post-questionnaire was administered immediately post the APP education session.

**Results:**

Health professionals found that APP was easy to navigate and culturally appropriate. Among the 80 carers, most were mothers (86%), aged between 26 and 50 years (75%) and 61% lived in remote settings (>100 km from a tertiary hospital). Most carers chose English audio (76%) with the remainder choosing one of the First Nations languages. Overall, asthma knowledge significantly improved post-education (median scores pre = 21 [interquartile range (IQR), 19–22; post = 24 (IQR 22–24), *p* = 0.05].

**Conclusion:**

The First Nations-specific multi-lingual Asthma APP was easy to use and acceptable for the use by health professionals that also significantly improved short-term asthma knowledge among First Nations carers of children with asthma. The Asthma APP is an innovative and culturally acceptable method of delivering evidence-based, health education to culturally and linguistically diverse populations among Australian First Nations people.

## Introduction

Asthma is a common respiratory condition affecting 1 in 9 Australians (1) with a prevalence 1.6 times higher among First Nations than non-First Nations Australians (2). Australian First Nations people are almost two times as likely to report asthma (3), have worse morbidity, and are three times more likely to die from asthma than non-First Nations people (4). Multiple factors likely contribute to this burden, such as exposure to tobacco and campfire smoke, chest infections, and poor asthma-related knowledge and management (5). It is well recognized that education is a key element in improving health-related outcomes (6, 7). However, the delivery of education to linguistically and culturally diverse groups where English is not often the first language spoken remains limited and needed. We have previously shown that culture-specific education programs are superior to generic ones to improve asthma-related outcomes (1, 3) and pictorial-based flipcharts improved short-term knowledge of carers of children with common childhood respiratory conditions, such as bronchiolitis, pneumonia, and chronic suppurative lung disease/bronchiectasis (4).

Yet, these one-to-one educational tools are time consuming, and accessibility beyond health facilities in rural and remote settings is often limited. Therefore, improving the method of delivery of health education to culturally and linguistically diverse populations that is innovative and culturally relevant (4, 8, 9), particularly for First Nations people (10) in remote settings where health staff turnover is high (11) and access to specialist services often limited is needed. One such mechanism is the use of digital platforms, such as mobile applications (APP), which have the capacity to improve access to health education in rural and remote settings (12).

Over the last decade, an increasing number of mobile health (mHealth) APPs have been developed globally to improve health-related outcomes for conditions, such as urinary incontinence (5), diabetes (13), anger problems (8), and hepatitis B knowledge (9) of inhalation therapy for asthma among health professionals (14). Yet, to the best of our knowledge, no mobile APP has been specifically designed for First Nations people with voice-over in local languages for common respiratory conditions. The use of mobile APPs as an innovative method of delivering health education in the current era is further supported by the increasing use and availability of network coverage in rural and remote locations of Australia. In our previous randomized controlled trial, we used mobile phones to follow-up First Nations children who lived in remote communities of the Northern Territory, where we achieved a follow-up rate of >90%; thus, the likelihood of a mobile APP as an effective educational modality is possible (15).

Our earlier study showed that carers had very poor baseline respiratory health knowledge and these flipcharts improved the knowledge of common respiratory conditions among First Nations carers (15). These carers appreciated the one-to-one education sessions and requested digital versions of these flipcharts (e.g., an APP) with an interactive voice-over in local languages (15). In response to this request and recommendations from Asthma Australia to provide culturally secure education (10) to First Nations people, we thus developed a First Nations-specific, multi-lingual, digital Asthma APP, based on our current pediatric pictorial asthma flipchart. In this study we, (a) assessed the functionality and usability of the Asthma APP with First Nations health professionals with and without asthma and (b) evaluated whether the Asthma APP improved knowledge and understanding of asthma among First Nations carers of children with asthma.

## Materials and methods

### Design and development of the APP

**Figure 1:**
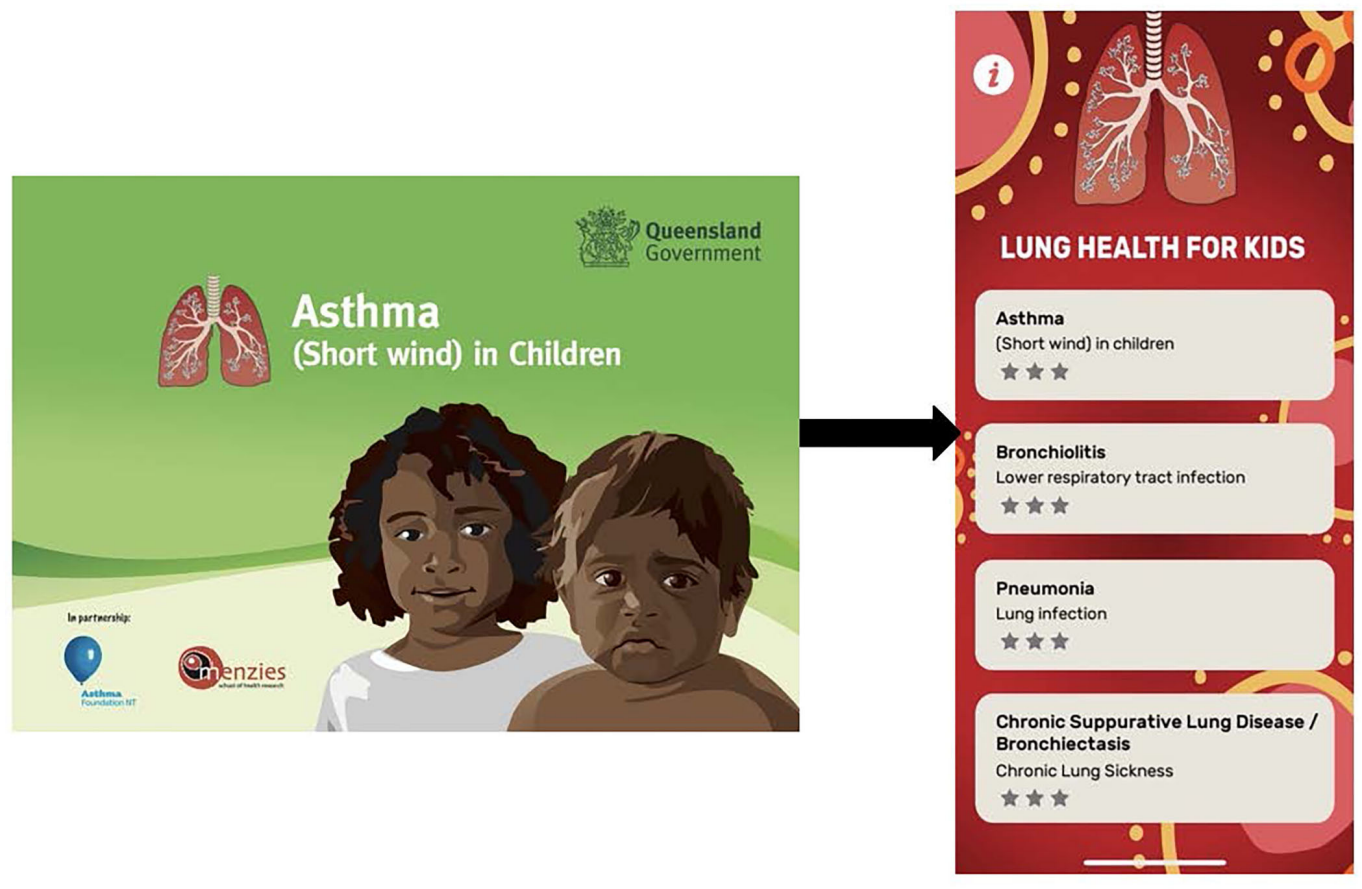
Transition from paper-based asthma flipchart to the “Lung Health for Kids APP”.

**Figure 2:**
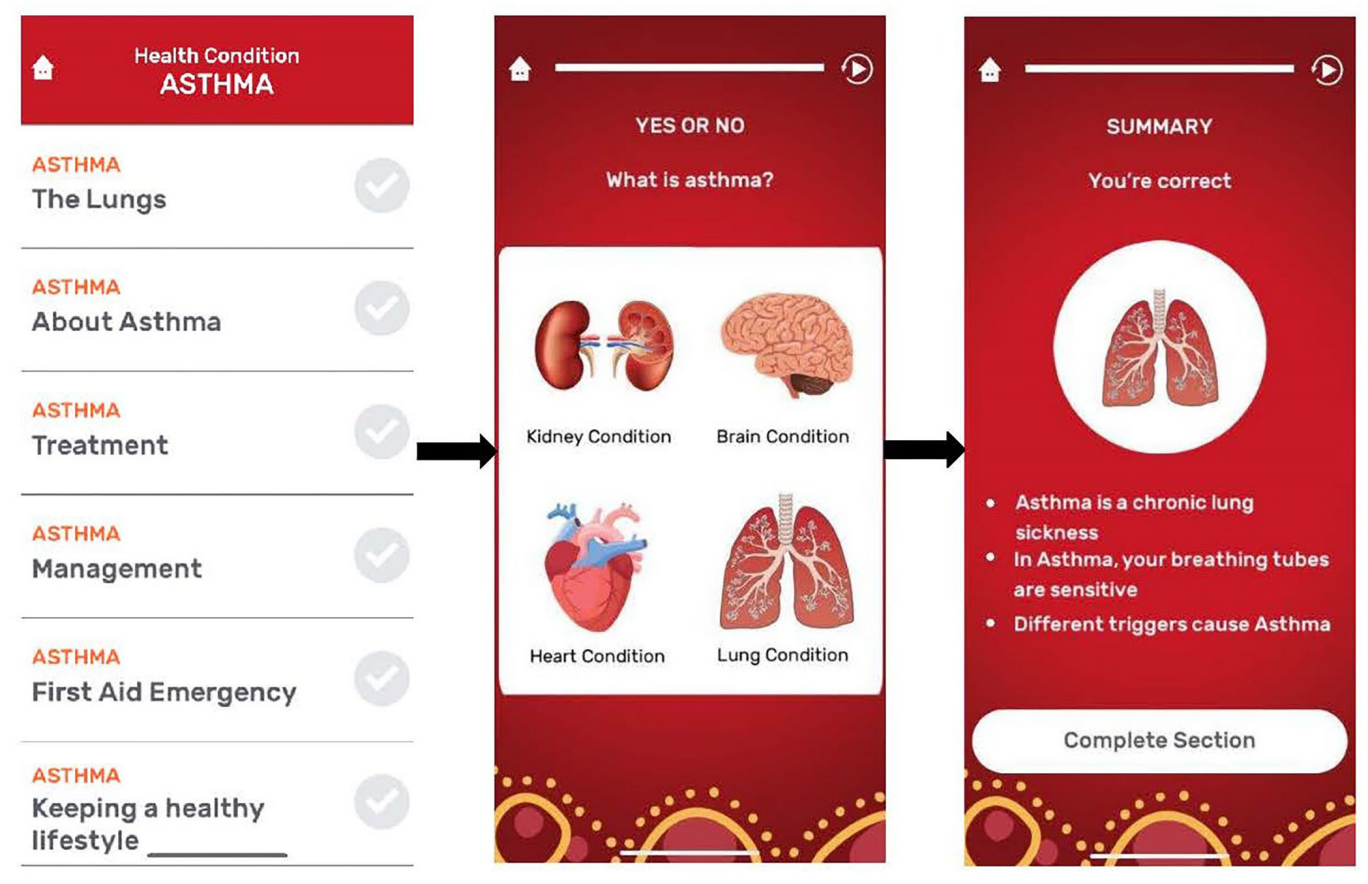
Component sections of Asthma APP.

A team of respiratory research nurses (that included a First Nations nurse) and medical professionals experienced working with First Nations people firstly mapped out the Asthma APP agreeing on the framework, functionality, and content based on our previous culture-specific pictorial-based asthma flipchart (Figure 1). The APP was separated into six sections that included (1) the lungs; (2) about asthma; (3) treatment; (4) management; (5) first aid emergency, and (6) keeping a healthy lifestyle, with up to 8 graphics cards using a mixture of static and interactive images, a brief content-specific explanation, and a short quiz (Figure 2).

A plain language storyboard script mirroring each graphics card was then written by our team and approved for readability, accuracy, and cultural appropriateness by members of the Menzies School of Health Research Australian First Nations Reference Group for Child Health and First Nations Health professionals. Linguists, from the Northern Territory Aboriginal Interpreter Service, further translated the storyboard script that was approved prior to audio files being recorded to ensure appropriateness for First Nations people. English and 7 First Nations languages from northern and central Australia included Tiwi, Murrinh-Patha, Yolngu Matha, Kriol, Warlpiri, Central Arrernte, and Pitjantjatjara. Forward and backward translation of the audio files were undertaken by the Aboriginal Interpreter Service to ensure accuracy prior to releasing these to the APP developer (Branium Labs). The audio files were then matched with the corresponding graphics card, and user testing of the APP was undertaken by health professionals and non-health professionals for usability, functionality, translation issues (where able), and to identify any glitches or further modifications prior to the evaluation component of our study.

### Health professional's evaluation of the APP

In total, 7 Australian First Nations Health Professionals with or without asthma from the Northern Territory were invited to undertake a semi-structured interview that focused on the functionality, usability, and general feedback of the APP. After written consent, baseline demographics were recorded on standardized data forms. Health professionals were then asked to review the APP in their own time, followed by a phone or face-to-face interview within 2 weeks of consent.

### Carer's evaluation of the APP

In total, 80 First Nations carers of children with a diagnosis of asthma who were recruited from pediatric respiratory outpatient clinics, during hospitalization, or through known contacts within our team from Darwin and Queensland, Australia were invited to evaluate the APP. Carers of children with bronchiectasis or cystic fibrosis were excluded. Carers were recruited from respiratory clinics, hospitals, social media, or word and mouth. The study was approved by the Human Research Ethics Committee of the Northern Territory Department of Health and Menzies School of Health Research (HREC 17-2919), the Children's Health Queensland Hospital and Health Service Human Research Ethics Committee (200219), and the Menzies School of Health Research Australian First Nations Reference Group for Child Health.

After written informed consent was obtained from the carer, baseline demographics were recorded on standardized data forms. Carers then underwent a pre-education questionnaire on asthma knowledge, followed by education using the Asthma APP, and then a post-education questionnaire immediately after. Carers had the option of working through the Asthma APP at their own pace with their local language, English, or without audio. The questionnaire was divided into six sections mirroring the APP, which included 25 multiple choice questions (maximum possible score of 25). Pre- and post-questionnaires were completed with one of our research nurses, followed by open-ended yarning questions about the usability and functionality of the APP and whether carers would recommend its use. Where possible, we repeated the post-questionnaire 2 weeks later with carers from Darwin.

Based on our previous flipchart study, our sample size was n = 20 for each flipchart (total n = 60), where we reported a *p* < 0.05 for 12 out of 23 questions from our questionnaires (4). In this study, our sample size of n = 80 provided 92% power to detect a 20% difference in pre- and post-education (assuming discordant proportions of 5 and 25%).

Data were entered on REDCap and analyzed using Stata v15.1 (StataCorp, College Station, TX, USA). Descriptive analyses are presented as median (interquartile range, IQR: 25th−75th) for continuous variables, while categorical variables are reported as counts and percentages. A chi-square test was used to compare categorical data. Total scores and then language groups were added together and compared in pre- and post-education using paired Wilcoxon Signed Rank test. A two-tailed *p*-value of < 0.05 was considered significant. The qualitative data were transcribed and the demographic data that included asthma status were recorded and then responses to specific questions about the design, usefulness and strengths, and weaknesses of the APP were organized into themes and then presented in a summary format.

## Results

### Health professional's perspective of the APP

In total, 7 First Nations health professionals who included four health practitioners, a pediatrician, a nurse, and a research assistant, participated in semi-structured interviews. Participants were aged between 31 and 50 years, most were women (71%) and four (57%) had asthma and a family history of asthma (57%), respectively. All participants spoke English at home and had a tertiary-level education. Five (71%) had previously used an APP in some capacity in their previous professional experience. Overall, the Health Professionals who assessed the APP found it to be useful, easy to navigate, and culturally appropriate; the compact portability of the APP and its colorful, engaging design, and lack of jargon were all seen as strengths. Several weaknesses included the monotones of several voice-overs, the lack of emergency numbers in remote areas, and two male participants commented that it's usefulness may be dependent on the situation and patient.

### Carers demographics, evaluation of the APP, and open-ended questions

The characteristics of 80 First Nations carers are presented in Table 1. Briefly, most were mothers (85%), aged between 20 and 50 years (86%), from a remote community (61%), with a family history of asthma (73%). Secondary education was relatively low; only 33% had completed high school and 21% had a tertiary qualification. Most carers chose English as the preferred language (76%), followed by Kriol (11%), Tiwi (6%), Yolngu Matha (4%), and Murrinh Patha (3%). No carers chose languages from Warlpiri, Central Arrernte, or Pitjantjatjara.

**Table 1:** Baseline demographics of child and carers.

**Variables**	**Total**
	**n** **=** **80 (%)**
**Child's details**	
Median (IQR) age (years)	6.5 (3.9–11.8)
Male	52 (65)
**Ethnicity**	
Aboriginal	53 (66)
Torres Strait Islander	14 (18)
Aboriginal and Torres Strait Islander	13 (16)
Remote*	49 (61)
**Site**	
Darwin	56 (70)
Queensland	24 (30)
**Primary carers details**	
**Relationship to child**	
Mother	68 (85)
Father	3 (4)
Grandparent	2 (3)
Aunty	3 (4)
Other	4 (5)
Female carers	75 (94)
**Age group of carers (years)**	
15–19	2 (3)
20–25	10 (13)
26–30	13 (16)
31–40	28 (35)
41–50	19 (24)
51–60	6 (8)
>60	2 (3)
**Language used in the APP**	
English	61 (76)
Tiwi	5 (6)
Kriol	9 (11)
Murrin Patha	2 (3)
Yolngu Matha	3 (4)
**Years carer completed at school**	
Primary school	5 (6)
Year 10	22 (28)
Year 11	9 (11)
Year 12	26 (33)
Tertiary qualification (completed)	17 (21)
Carers with asthma	22 (28)
Family members with asthma	58 (73)

IQR, interquartile range.

* >100 km from tertiary hospital.

Overall asthma knowledge was significantly improved after the APP; the pre-questionnaire median score was 21 (IQR 19–22) to post-questionnaire = 24 (22–24), *p* = 0.05. When assessing specific languages that for the English audio (n = 61) remained significant [pre-questionnaire = 21 (IQR 18–22); post-questionnaire = 23 (22–24), *p* < 0.0001] while that for a First Nations language (n = 19) were not significant [pre-questionnaire = 21 (IQR 15–22), post-questionnaire = 23 (21–24), *p* = 0.3)].

There were significant improvements in some individual questions' pre- and -post questionnaires, particularly for asthma first aid (Table 2) and the type of language used (Table 3). Overall, all but one carer found the APP fun and interactive and recommended its use. The one carer who did not recommend the APP did not provide any feedback. The most common responses from the open yarning questions are summarized in Table 4.

**Table 2:** Comparison of pre- and post-questionnaire scores from carers.

**Questions by section**	**Number (%) of carers who answered the question correctly**		
	**Pre**	**Post**	***p*-value**
	**n = 80 (%)**	**n = 80 (%)**	
**About the lungs**			
Number of lungs?	71 (89)	79 (99)	0.008
What do the lungs do?	67 (84)	74 (93)	0.07
Is oxygen important?	80 (100)	80 (100)	1.0
**About asthma**			
Do you know what asthma is?	66 (83)	78 (98)	0.002
What part of the body does asthma effect?	75 (94)	79 (99)	0.13
Are breathing tubes in asthma more sensitive?	55 (69)	79 (99)	<0.0001
Signs and symptoms of asthma	78 (98)	79 (99)	1.0
Triggers for asthma	69 (86)	74 (93)	0.06
**Asthma treatment**			
Treatments for asthma	70 (88)	77 (96)	0.02
Are asthma action plans important?	77 (96)	78 (98)	1.0
Is a spacer important to use with a puffer?	80 (100)	80 (100)	1.0
What does a reliever puffer do?	72 (90)	78 (98)	0.07
What do preventer puffers do?	57 (71)	69 (86)	0.01
**Asthma management**			
How often should my child see a doctor?	72 (90)	78 (98)	0.03
How to tell if child's asthma is OK	63 (79)	70 (88)	0.09
How to tell if your child has a bit of short wind	69 (86)	71 (89)	0.8
How do you know if your child has bad short wind?	73 (91)	75 (94)	0.6
When should you call an ambulance ‘000'?	71 (89)	76 (95)	0.1
**Asthma first aid emergency**			
First step in asthma first aid	37 (46)	74 (93)	<0.0001
Second step in asthma first aid	59 (74)	77 (96)	<0.0001
How long should you wait if no improvement?	24 (30)	67 (84)	<0.0001
What to do if your child is not improving	61 (76)	73 (91)	0.001
What do you while waiting for an ambulance?	60 (75)	68 (85)	0.06
**Keeping a healthy lifestyle**			
How often should I take my child for check-ups?	66 (83)	70 (88)	0.3
What will keep my child's lungs healthy?	74 (93)	74 (93)	1.0

**Table 3:** Comparison of pre- and post-questionnaire scores from cares by language used.

**Questions by section**	**Number (%) of carers who answered the question correctly**					
	**First Nations language**			**English language**		
	**Pre**	**Post**	***p*-value**	**Pre**	**Post**	***p*-value**
	***n* =19 (%)**	***n* = 19 (%)**		***n* = 61 (%)**	***n* = 61 (%)**	
**About the lungs**						
Number of lungs	16 (84)	18 (95)	0.5	55 (90)	61 (100)	0.03
What do the lungs do?	12 (63)	16 (84)	0.3	55 (90)	58 (95)	0.3
Is oxygen important?	19 (100)	19 (100)	1.0	61 (100)	61 (100)	1.0
**About asthma**						
Do you know what asthma is?	14 (74)	19 (100)	0.06	52 (85)	59 (97)	0.04
What part of the body does asthma effect?	15 (79)	18 (95)	0.3	60 (98)	61 (100)	1.0
Are breathing tubes in asthma more sensitive?	9 (47)	18 (95)	0.004	46 (75)	61 (100)	0.0001
Signs and symptoms of asthma	18 (95)	18 (95)	1.0	60 (98)	61 (100)	1.0
Triggers for asthma	15 (79)	17 (89)	0.5	54 (89)	57 (93)	0.3
**Asthma treatment**						
Treatments for asthma	14 (73)	17 (89)	0.3	56 (92)	60 (98)	0.1
Are asthma action plans important?	17 (89)	18 (95)	1.0	60 (98)	60 (98)	1.0
Is a spacer important to use with a puffer?	18 (95)	19 (100)	1.0	61 (100)	61 (100)	1.0
What does a reliever puffer do?	16 (84)	17 (89)	1.0	56 (92)	61 (100)	0.06
What do preventer puffers do?	11 (58)	15 (79)	0.3	46 (75)	54 (89)	0.04
**Asthma management**						
How often should my child see a doctor?	16 (84)	17 (89)	1.0	56 (92)	61 (100)	0.06
How to tell if your child's asthma is OK?	15 (79)	17 (89)	0.5	48 (79)	53 (87)	0.2
How to tell if your child has a bit of short wind?	15 (79)	16 (84)	1.0	54 (89)	55 (90)	1.0
How to tell if your child has bad short wind?	15 (79)	17 (89)	0.5	58 (95)	58 (95)	1.0
When should you call an ambulance ‘000'?	15 (79)	18 (95)	0.3	56 (92)	58 (95)	0.6
**Asthma first aid emergency**						
First step in asthma first aid	10 (53)	18 (95)	0.008	27 (44)	56 (92)	<0.0001
Second step in asthma first aid	12 (63)	19 (100)	0.02	47 (77)	58 (95)	0.003
How long should you wait if no improvement?	7 (37)	13 (68)	0.03	17 (28)	54 (89)	<0.0001
What to do if your child is not improving?	12 (63)	16 (84)	0.2	49 (80)	57 (93)	0.008
What do you while waiting for an ambulance?	11 (58)	14 (74)	0.4	49 (80)	54 (89)	0.2
**Keeping a healthy lifestyle**						
How often should I take my child for check-ups?	15 (79)	16 (84)	1.0	51 (84)	54 (89)	0.5
What will keep my child's lungs healthy?	16 (84)	16 (84)	1.0	58 (95)	58 (95)	1.0

**Table 4:** Carers opinion of the Asthma APP.

**Common questions**	**Examples of responses from carers**
Did you learn anything more about asthma using the APP?	Treatments, types of puffers, use of spacers, function of the lungs, asthma first aid [particularly sitting upright and waiting time (4 mins), reinforcing management of asthma]
Most important things to remember from the APP?	Function of the lungs, what to do in asthma first aid, using an asthma action plan, ensuring management is maintained, using the right puffers with spacers

No carer was available to undertake another post-questionnaire 2 weeks after enrolment.

## Discussion

In this study, we described the process of developing and evaluating an innovative, First Nations-specific, multi-lingual, digital Asthma APP. Of the 7 First Nations health professionals who assessed the functionality and usability of the APP, they found this to be an engaging, easy-to-use tool that provided culturally appropriate, evidence-based asthma education to First Nations families of children with asthma. While some constructive feedback was noted, all health professionals recommended that the APP would be a valuable tool to accompany the clinical practice. We also showed that among 80 First Nations carers of children with asthma from the Northern Territory and Queensland, short-term asthma knowledge significantly improved after the use of the Asthma APP, particularly for some individual questions, such as how asthma is treated, first aid of assessed, and by the language used.

With improved accessibility of smartphones over the last decade, the number of mHealth APPs has steadily grown globally, as they are a low-cost resource, can be used at any time, in any geographical location (16), and have been shown to be an acceptable and easy modality for communicating with health care providers and improving health-related outcomes for patients with chronic conditions. For example, in children and adolescents with cancer, a systematic review found that the use of mHealth APPs was a suitable method for patients and families to access reliable, up-to-date education that provided better communication between patients and health providers (17). Importantly, in settings where English is often a second or third language, differences in language and cultural understanding often result in communication gaps between health providers and patients often impacting poorer health outcomes (18). To increase awareness and understanding of asthma to improve asthma management and health-related outcomes, peak national (19) and international (20) asthma bodies recommend tailoring asthma education that is culturally appropriate, particularly among resource-poor settings and culturally and linguistically diverse populations e.g., Australian First Nations.

Nevertheless, of the ≈1,500 APPs targeting various aspects of asthma (e.g., compliance, symptom, medication tracking, improving inhaler techniques, health education, text messaging, interactive websites, and the environment) on Google Play and the Apple stores, none are available for Australian First Nations children (21). The lack of culture-specific mHealth APPs for First Nations people was further highlighted in a systematic review in 2019 on mHealth APP interventions, whereby only 13 studies from Australia, New Zealand, and the United States, covering conditions, such as sexual health, fatherhood, suicide, and heart failure patient support, were available (22). Of the two mHealth APPs (23, 24) identified in this review, both were from Australia for mental health that used visual pictures, action-based content, and audio voice-overs, yet local First Nations languages were not used. We did, however, find a sole study from Arnhem Land in the Northern Territory that developed a bilingual APP for hepatitis B upon request from local health staff and educators that was culturally appropriate, audio-visual, interactive, and upon initial evaluation demonstrated a significant improvement in hepatitis B knowledge after reviewing the APP (9).

To the best of our knowledge, our study contributes for the first time novel data on an innovative method for providing health education for childhood asthma using a First Nations-specific Asthma APP with voice-over in 7 local languages and English. The strengths of our study include that our APP was in response to requests from consumers, i.e., our First Nations families. Second, the design and development were in collaboration with our Australian First Nations Reference Group for Child Health and First Nations Health professionals across multiple sectors. Third, we used culturally appropriate pictures previously used on our paper-based flipchart on each card with a short plain language description. Importantly, pictures have been previously shown to enhance learning and easier recall of information and can facilitate word memory (25). Fourth, we used the Northern Territory Aboriginal Interpreter Service to convert our asthma storyboard script into local languages, who then audio recorded the script and provided forward and backward translation to ensure accuracy of the recordings. Fifth, we were able to replicate the findings of our original flipchart-evaluation study that short-term asthma knowledge increased (4). We were, however, unable to demonstrate longer-term asthma knowledge as no carer was able to undertake the post-questionnaire 2 weeks after the baseline visit as most lived in remote communities of the Northern Territory. Finally, the advantages of our APP are that additional languages and up-to-date evidence can be added relatively quickly to ensure that accurate health information is accessible to people, particularly in regions where access to specialist services is limited and the turnover of staff is high (11).

Despite these many strengths, our study also has limitations. Firstly, creating a storyboard in plain English without losing the accuracy of medical terminology was challenging. Not all words could be translated word-to-word from English to First Nations languages or word to a cultural meaning, thus we had to work closely with linguists to find words that would be acceptable to users of the APP from each language group (e.g., some words remaining in English). Secondly, despite having processes in place for forward and backward translation with the Aboriginal Interpreter Service, we found in some language groups, old and new languages were spoken within communities, thus we needed to re-record some of the audio languages to maintain accuracy. Thirdly, we were limited in the number of First Nations languages we could include in the APP due to funding limitations. We thus chose language groups with the widest reach from geographically diverse regions across the Northern Territory. Fourth, in the evaluation component of the study, we were unable to recruit carers from central Australia or beyond specialist clinics or the hospital, thus it remains unknown the effectiveness of the APP in remote communities. While this was not ideal, we do not, however, think this would have impacted our results, as overall asthma knowledge was improved when carers used English or when using a language. Lastly, only health professionals from the Northern Territory were included in the qualitative assessment, thus we cannot be certain of the impact regional differences may have had on our outcomes. Further evaluation of the APP in clinical practice outside of respiratory outpatient clinics and hospitalization, however, should be ongoing to ensure the effectiveness of this innovative educational tool. Nevertheless, our APP has been widely disseminated and embedded across training programs, e.g., Queensland Aboriginal Health Worker Training, Northern Territory Primary Care Information System, Central Australian Rural Practitioners Association, Remote Primary Health Care Treatment Manual, and other First Nations educational repositories, and is free to download from the Apple Store (https://apps.apple.com/au/app/lung-health-for-kids/id1509172445) or Google Play (https://play.google.com/store/apps/details?id=com.menzies.lungapp1&referrer=utm_source%3Dgoogle%26utm_medium%3Dorganic%26utm_term%3Dlung+health+for+kids+app).

In summary, this First Nations-specific multi-lingual Asthma APP was easy to use and acceptable for the use by health professionals that also significantly improved short-term asthma knowledge among First Nations carers of children with asthma. The Asthma APP is an innovative and culturally acceptable method of delivering evidence-based, health education and provides a platform to broaden other respiratory conditions or chronic conditions for Australian First Nations children and adults.

## Data availability statement

The datasets presented in this article are not readily available because as per our institutions' policies involving Australian First Nations children and in accordance with national guidelines, we are unable to share individual participant data as specific consent for this was not obtained. Requests to access the datasets should be directed to gabrielle.mccallum@menzies.edu.au.

## Ethics statement

The studies involving human participants were reviewed and approved by Human Research Ethics Committee of the Northern Territory Department of Health and Menzies School of Health Research (HREC 17-2919) and Children's Health Queensland Hospital and Health Service Human Research Ethics Committee (200219). Written informed consent to participate in this study was provided by the participants' legal guardian/next of kin.

## Author contributions

GM and AC conceived the study and participated in the study design. GM developed the protocol, data collection forms, database, coordinated the study from inception to analysis, and oversight of manuscript preparation by LV. LV, CW, and FT actively recruited participants. GM had full access to the raw data and conducted the statistical analysis for the quantitative component. SC conducted the analysis for the qualitative component. GM and LV drafted the initial manuscript with substantial revisions undertaken by GM. All authors contributed to editing of the manuscript and approved the final version.

## Funding

This study was funded by Asthma Australia, a National Health and Medical Research Council Centre for Research Excellence in Lung Health (Grant 1040830; www.crelungs.org.au), and Queensland Health. ABC was supported by an NHMRC practitioner fellowship (Grant No. 1154302).

## Conflict of interest

The authors declare that the research was conducted in the absence of any commercial or financial relationships that could be construed as a potential conflict of interest.

## Publisher's note

All claims expressed in this article are solely those of the authors and do not necessarily represent those of their affiliated organizations, or those of the publisher, the editors and the reviewers. Any product that may be evaluated in this article, or claim that may be made by its manufacturer, is not guaranteed or endorsed by the publisher.
